# An independent evaluation of laboratory staffing needs: Launching the forensic laboratory workforce calculator

**DOI:** 10.1016/j.fsisyn.2021.100137

**Published:** 2021-02-12

**Authors:** Paul J. Speaker

**Affiliations:** John Chambers College of Business and Economics, West Virginia University, Morgantown, WV, 26505, USA

**Keywords:** Workforce, Backlogs, Economies of scale, Efficiency

## Abstract

The 2019 NIJ Report to Congress on the needs of the forensic science community [1] highlighted the staffing deficit of forensic scientists by more than 900 positions. The Report emphasized the impact of the opioid crisis and the evolution of synthetic opioids on the demands for forensic laboratories. The resource drain attributable to the opioid crisis has filtered into all other areas of investigation as laboratories divert limited resources from other uses to meet the high demand in drug chemistry and toxicology from opioid abuse. We introduce the forensic laboratory workforce calculator, a tool that any forensic laboratory may use to evaluate their current personnel allocation and estimate any under- or over-staffing to meet current or estimated caseloads. The forensic laboratory workforce calculator is available free to any laboratory through the Forensic Technology Center of Excellence website.

## 1 Introduction^1^

The 2019 NIJ Report to Congress on the needs of the forensic science community [1] highlighted the staffing deficit of forensic scientists of more than 900 positions to serve the nationwide caseload faced by state and local forensic laboratories. The Report emphasized the impact of the opioid crisis and the evolution of synthetic opioids on the demands for forensic laboratories and the resulting increases in turnaround times and the corresponding growth in the backlog of casework across all areas of investigation. The resource drain attributable to the opioid crisis has seen an increased demand for laboratory resources in drug chemistry and toxicology. That resource drain has filtered into all other areas of investigation as laboratories divert limited resources from other uses to meet the high demand in drug chemistry and toxicology from opioid abuse. The workforce difficulties during the COVID-19 pandemic have compounded the workforce challenges. In an effort to meet this growing demand in casework examination for drugs-controlled substances, toxicology ante-mortem, and toxicology post-mortem, laboratories responded with increases in productivity [[2], [3], [4]]. However, the higher productivity by existing operational staff has not been enough to keep up with the increased demand for services.

The societal cost from the delays in casework investigation has a devastating impact on the U.S. economy. The Council of Economic Advisors estimates that the cost to the U.S. economy from the opioid crisis may exceed $600 billion dollars per year [5]. This societal cost represents over two percent of U. S. Gross Domestic Product. This loss includes costs from medical care, addiction treatment, justice system processing, lost productivity, and loss of life [[6], [7], [8], [9], [10], [11], [12], [13]]. To reduce this impact and the associated portion of those societal costs to state and local criminal justice systems, forensic laboratories need more resources allocated to fill those 900 plus deficit positions. Unfortunately, the Report to Congress [5] only presents the macro need and does not offer the means to measure the need for positions at the individual jurisdiction or laboratory level. We created the forensic laboratory workforce calculator as an objective tool to independently assess an individual laboratory’s staffing needs.

Forensic laboratories exhibit similar output capabilities as demonstrated in the provision of all goods and services. There are economies of scale associated with the examination of forensic evidence, which makes the allocation of the 900 plus shortage of full-time equivalent employment (FTE) a complex issue. Fortunately, economic theory and econometric analysis permit the estimation of those needs at the individual laboratory level.

In this manuscript, we introduce the forensic laboratory workforce calculator, a tool that any forensic laboratory may use to evaluate their current personnel allocation and estimate any under- or over-staffing to meet current or estimated caseloads. The underlying model takes advantage of twelve years of data from Project FORESIGHT [14,15] and a decade of research using that data. In the next section, we describe Project FORESIGHT and the FORESIGHT data. Following that section, we present the economic rationale for the efficiency measures and the corresponding efficient frontier. From those efficiency measures, the American Society of Crime Laboratory Directors (ASCLD) Maximus award selection is outlined as the criteria for the selection of exemplars for the underlying calculator equations. Next, we review the beta test process and the changes made to the enhanced calculator following the suggestions from testing laboratories. Next, the inputs and output for the calculator are presented. A brief discussion and concluding comments follow.

## 2 Project FORESIGHT

The data underlying the construction of the workforce calculator comes from Project FORESIGHT [14,15]. Project FORESIGHT collects annual data from forensic laboratories across the world. Laboratories provide data on casework, financials, and personnel allocation. Data submission includes individual laboratories and laboratory systems that complete the Laboratory Reporting and Analysis Tool (LabRAT). Note that Project FORESIGHT data does not come from a random sample of forensic laboratories. While Project FORESIGHT invites all forensic laboratories around the world to submit data, the data submitted to Project FORESIGHT are voluntary submissions. The number of participating laboratories, or laboratory systems, has grown from the initial 17 laboratories in fiscal year 2008 to 188 in fiscal year 2019.

Project FORESIGHT collects data on two levels of detail. Level I data represents the minimum detail on caseload, FTE, and expenditures required for economic analysis. Level II data expands upon the caseload detail with information on items examined internally and outsourced, samples examined internally, tests performed, reports written, turnaround time, and backlogs.^2^ Level II financial detail includes capital, consumable, and overhead expenditures allocated to the specific areas of investigation. Since Level II data falls beyond the minimum submission detail, the workforce calculator relied upon the Level I data to obtain the broadest representation of laboratories.

**Fig. 1:**
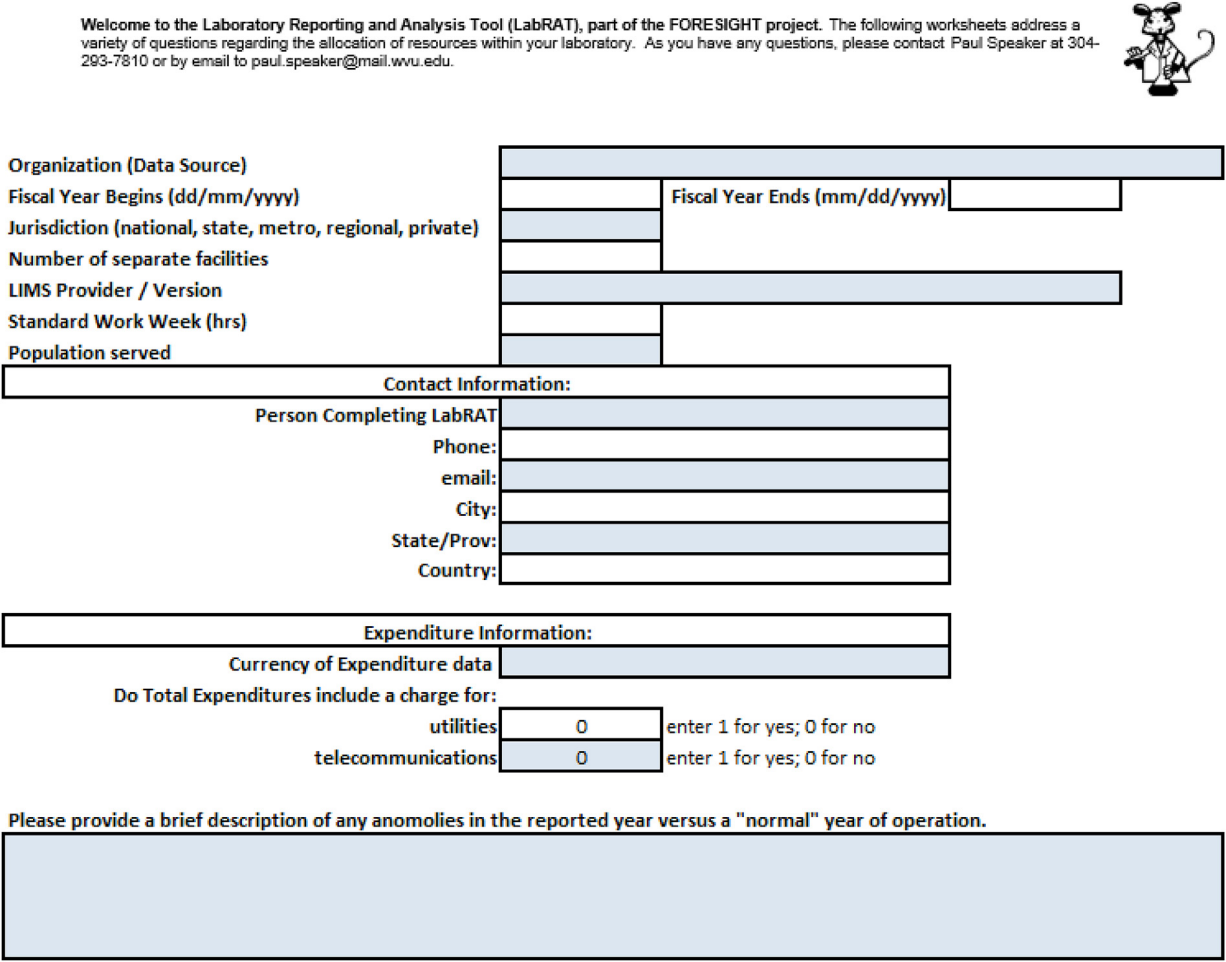
LabRAT opening page.

Level I submission includes the data highlighted in the following three figures. Fig. 1 represents the opening page of the LabRAT data submission tool. The page serves primarily as a contact information sheet. However, there are a few additional items requested beyond contact details. Each laboratory submits data that corresponds to the fiscal year as defined by its jurisdiction.

The two budgetary questions regarding direct charges for utilities and telecommunications ensure that all costs are included in the overhead charges in the expenditure worksheets. Many jurisdictions satisfy their obligations in these areas outside the direct budgets of the laboratories. In those cases, the FORESIGHT cost calculations assess an estimated cost for these necessities. Additionally, laboratories indicate any extraordinary situations to aid in the evaluation and interpretation of the performance by the laboratory.

**Fig. 2:**
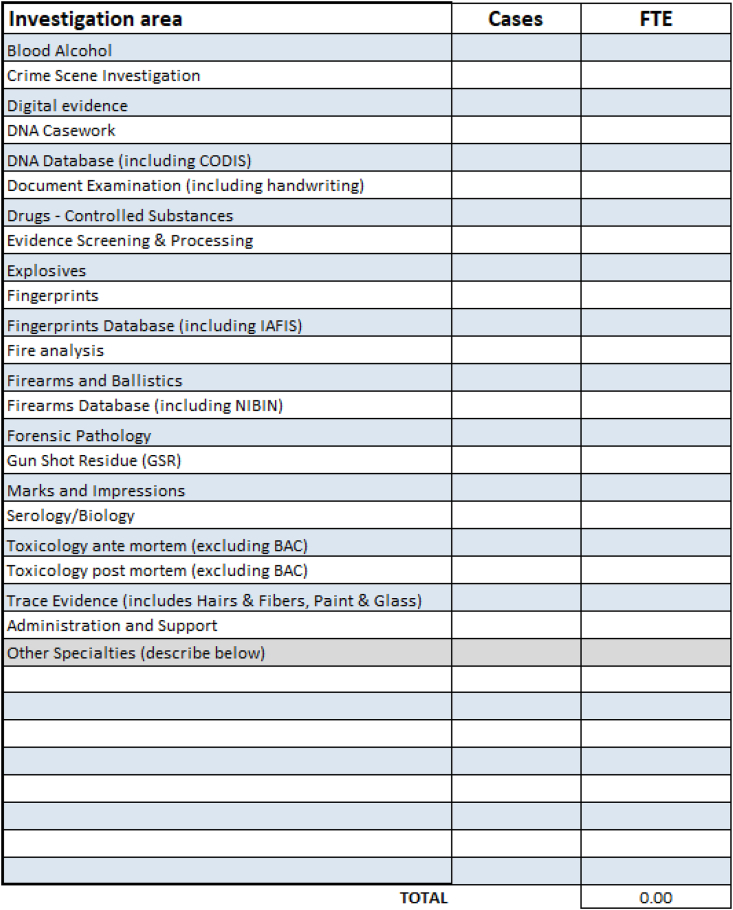
LabRAT level I casework.

Fig. 2 illustrates the minimum casework and personnel data submission. The definitions for the areas of investigation are included with the LabRAT tool for consistent interpretation. The caseload represents case submission over the time period indicated on the opening page, generally the fiscal year for the corresponding jurisdiction. The FTE include an assessment of the full-time equivalent for full-time, part-time, temporary, and overtime employees for filled positions. Note that the FTE indicated under Administration and Support are allocated to the individual areas of investigation as a weighted average of assigned FTE.

**Fig. 3:**
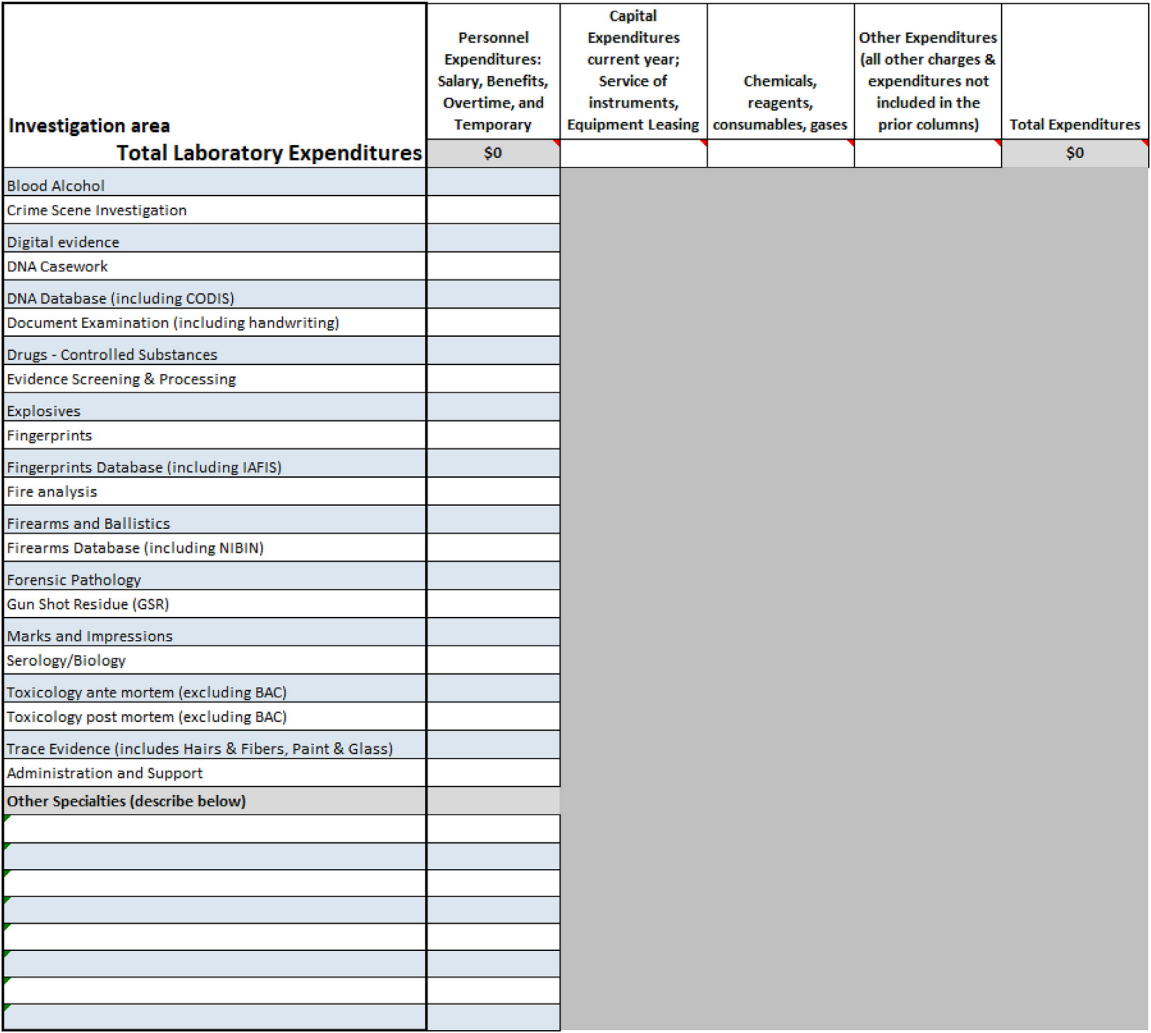
LabRAT level I expenditures.

Fig. 3 illustrates the minimum expenditure data required for FORESIGHT analysis. Personnel expenditures are required to be broken down to the individual areas of investigation. Generally, personnel expenditures represent the lion share of laboratory expenditures. Laboratory-wide allocations for capital, consumables, and overhead complete the detail needed to provide a report for each submitting entity.

## 3 The efficient frontier

Examination of the mission statements of forensic laboratories reveals some common themes. Synthesizing these mission statements into a unified objective statement suggests that laboratories attempt to analyze evidence correctly and process as many cases as budgets permit [16].^3^ The cases processed relative to the allocated budget can be broken down into a series of ratios that help to explain how a laboratory has been able to achieve its specific level of case throughput [17]. To illustrate, let CASE be the number of processed cases over a given time period and TOTEXP represent the total expenditures over that period for a given area of investigation. The ratio CASE/TOTEXP represents the metric to be maximized as a manifestation of the mission of the laboratory. A laboratory’s ability to increase its throughput for its given quality standards is affected by its internal policies and procedures, allocation of expenditures between long-term investment (capital expenditures) and immediate processing (personnel, consumables, and overhead expenditures), local economic forces, and productivity of its personnel. Let SMPL represent the number of samples examined in an area of investigation, PEREXP represent the corresponding total personnel expenditures, and FTE the associated full-time equivalent personnel. Then,

CASETOTEXP=(SMPLFTE)×(PEREXPTOTEXP)(SMPLCASE)×(PEREXPFTE)

$$=\frac{\left(Productivity\right)\times \left(\%\;Personnel\;Expenditure\right)}{\left(Sampling\;Intensity\right)\times \left(Average\;Compensation\right)}$$

This decomposition of the ratio of cases processed to total expenditures reveals that a laboratory’s performance may be explained by four ratio relationships. The two numerator ratios indicate that case processing occurs at a higher rate when the laboratory demonstrates greater throughput per person or when more expenditures are allocated towards personnel. This latter ratio reflects more immediate results versus investment in capital expenditures for higher long-term results. Additionally, increases in either denominator ratio will reduce cases processed for a given level of expenditures. This comes through greater sampling intensity or higher compensation levels in the laboratory relative to other laboratories.

**Fig. 4:**
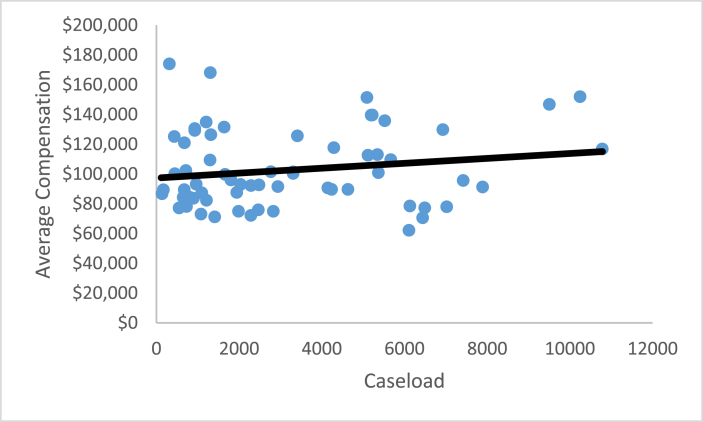
Average compensation versus caseload.

**Fig. 5:**
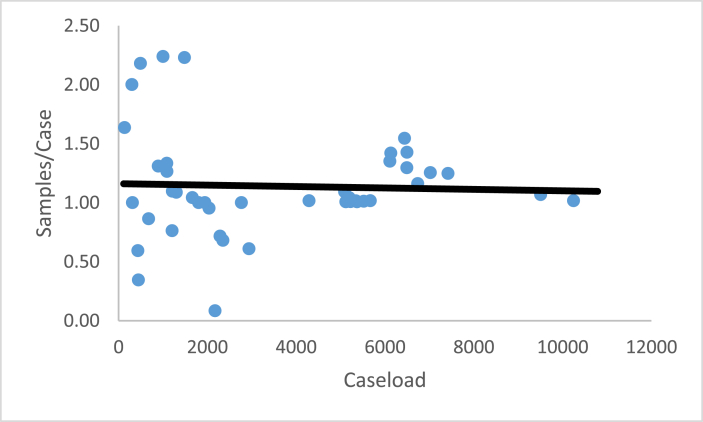
Samples per case versus caseload.

**Fig. 6:**
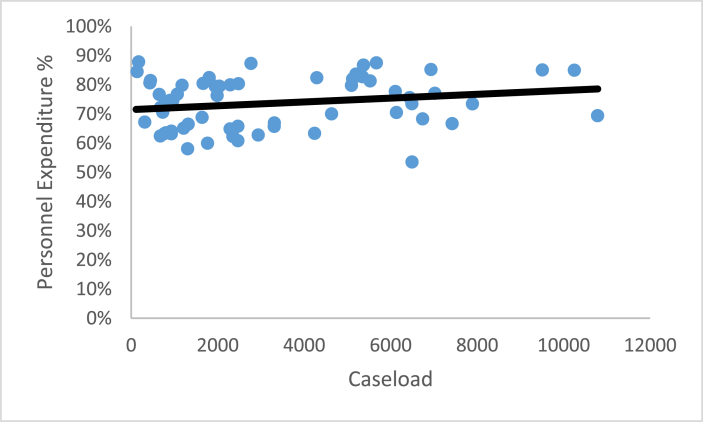
Personnel expenditure percentage.

These four ratios enable a laboratory to interpret their laboratory performance compared to other FORESIGHT laboratories. For three of the ratios, average compensation, sampling intensity, and percent personnel expenditures, a comparison of the laboratory’s performance relative to the benchmark FORESIGHT summary statistics provides a simple interpretation of the underlying forces behind the laboratory’s CASE/TOTEXP. Fig. 4, Fig. 5, Fig. 6 illustrate the relationship between these metrics and caseload for toxicology ante mortem.^4^

For each of these three figures, the solid line represents the results of a simple linear regression of the ratio relative to caseload. Fig. 4 shows a slightly increasing average compensation as the caseload increases. However, regardless of the caseload level, there is no particular pattern above or below the ordinary least squares regression line.

Fig. 5, sampling intensity versus caseload suggests a slightly negative relationship as caseload increases, while Fig. 6, personnel expenditure percentage versus caseload, shows the slightest positive slope in the linear regression. Caseload does not offer a good explanation as to why either ratio differs from benchmark laboratories. The personnel expenditure percentage for laboratories at every case level displays a similar range regardless of caseload.

**Fig. 7:**
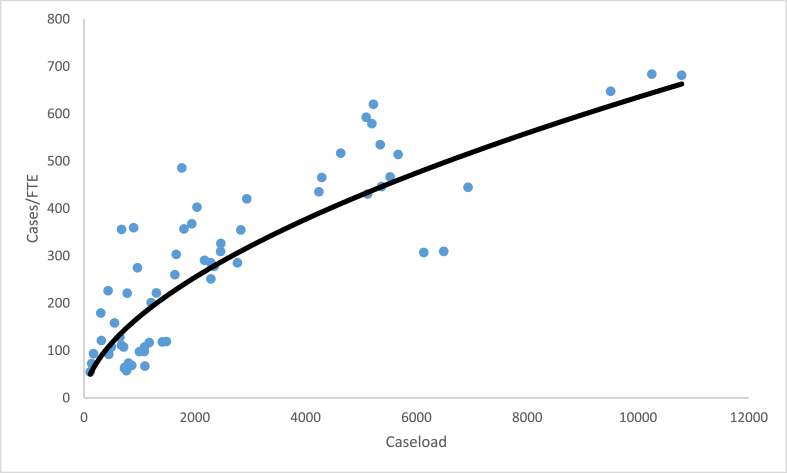
Relationship between throughput and caseload.

Fig. 7, productivity versus caseload, indicates a very different relationship between the ratio samples per FTE and the caseload. This is expected. The expected productivity within the laboratory bears a non-linear relationship with the size of the caseload submitted to the laboratory [[18], [19], [20], [21], [22], [23], [24], [25], [26]]. The estimation of the relationship does not appear to be linear. The curved line within the scatter plot in Fig. 7 is the result of a double logarithmic linear regression as a reflection of the productivity change expected to accompany economies of scale. For the illustrated area of investigation, productivity rises at a decreasing rate. Ultimately, a caseload would reach a level where productivity declines. However, no laboratory in the sample appears to have reached the size where productivity declines.

While Fig. 4, Fig. 5, Fig. 6, Fig. 7 illustrate the relationship between each of the four ratios and caseload, a similar relationship exists for each of the other twenty areas of investigation. That is, caseload explains very little of the variations in average compensation, sampling intensity, or the personnel expenditure percentage. However, for each area of investigation, economies of scale are in evidence as the relationship between productivity and caseload follows a non-linear relationship leading to an inverted U-shaped curve. For some areas, as with toxicology ante mortem analysis only the upward-sloped portion of the productivity curve is realized, while for other areas both the upward- and downward-sloped portions are observed.

## 4 The Maximus award criteria

The relationship between productivity and caseload highlighted in Fig. 7 repeats across every area of investigation. As caseloads increase, productivity will increase up to a point and eventually decrease as diseconomies of scale appear. Unlike a private sector business, where competitive forces move the level of activity towards perfect economies of scale, the characteristics of a political jurisdiction, such as population served and crime rates within the jurisdiction, determine the bounds of performance for a laboratory. That suggests that an evaluation of efficiency for each laboratory must be benchmarked against the productivity potential for a given caseload.

The American Society of Crime Laboratory Directors (ASCLD) recognizes high performing laboratories each year with the Maximus Award. The award recognizes laboratories that have performed at a 90% efficiency level or higher, based upon a weighted average of performance across areas of investigation. The Project FORESIGHT data collection separates activity into 21 areas of investigation. Although there are 21 categories, it is the rare laboratory that conducts evidence analysis in all areas. Individual laboratories have a jurisdictional mandate for which areas of investigation the laboratory provides analysis. The vast majority of forensic laboratories analyze data in six to twelve of these areas of investigation. Because of this variation in activity across laboratories, the efficiency metric considers each activity of the laboratory, determines the fully allocated expenditures for its caseload, and compares that expenditure to the estimation of the efficient frontier (i.e., the estimated curve as illustrated in Fig. 7). The deviations of actual cost from the estimated efficient cost is weighted by the percentage of total laboratory expenditures. The summed weighted percentage across all areas determines the metric.

The Maximus award criteria serve as the foundation for the estimation of the efficient frontier for each area of investigation. The high productivity performers are used to project the possibilities at all levels of casework to form the efficient frontier.

## 5 The beta workforce calculator

We created the beta workforce calculator as a proof of concept for laboratories to test their current caseload and staffing. We launched the first attempt in February 2019 with the intention to solicit feedback from the beta workforce calculator that would assist in the testing of the embedded concepts.

Initial questions in the development of the test calculator included considerations of the data available from Project FORESIGHT, Maximus award criteria, and external data that might influence laboratory productivity. External data included data from the FBI Uniform Crime Reporting (UCR) Program and population data from the U.S. Bureau of the Census.

Project FORESIGHT data included over one thousand submissions across investigative areas with the data categories highlighted in Fig. 2, Fig. 3. We limited our estimations of the relationship between staffing levels and optimal productivity to the high performing laboratories that met the qualifications for the Maximus award criteria.

For each area of investigation, we observed the scatter plots of the productivity metric, similar to Fig. 7, and estimated the non-linear regression suggested by the data for the relationship between productivity and caseload. In some areas, the scatter plot suggested the theoretical inverted U-shape and we estimated a quadratic regression with respect to caseload (caseload and caseload-squared) and a series of other independent variables (X_i_). The independent variables include (0,1) dummy variables for type of jurisdiction (metropolitan or state), population or natural logarithm of population, state violent crime rate, state property crime rate, and interactions between the dummy variables and the other independent variables.

CasesFTE=β0+β1Cases+β2Cases2+∑βiXi

In other areas of investigation, the scatter plot suggested that perfect economies of scale had yet to be reached. We considered two alternative specifications for the relationship between productivity and caseload, logarithmic and double logarithmic. In the logarithmic specification, we treated the natural logarithm of caseload as an independent variable.

CasesFTE=β0+β1Ln(Cases)+∑βiXi

In the double logarithmic specification, we use the natural logarithm of productivity as the dependent variable and the natural logarithm of caseload as an independent variable.

Ln(CasesFTE)=β0+β1Ln(Cases)+∑βiXi

For each of these specifications the series of other independent variables (X_i_), as used with the quadratic regression, were employed.

## 6 The enhanced workforce calculator

The second round of workforce calculator development benefited from two additional years of FORESIGHT data and the comments and suggestions of several test laboratories from the beta workforce calculator. We reviewed all laboratories in the Project FORESIGHT database using the Maximus award criteria. From those laboratories meeting the award standard for the weighted average performance, we reviewed performance within each area of investigation to identify those laboratories that met the 90% or higher efficiency standard for inclusion in the data for estimation of the efficient frontier.^5^ The expanded data set enabled a broader range of caseloads for consideration for regional, metro, and state jurisdictions.

In addition to the FORESIGHT data, we included population data from the U.S. Census Bureau and State-level data from the FBI’s Uniform Crime Reporting Program. The estimated efficient frontier for each area of investigation follows a non-linear estimation as described by economic theory and suggested by observation of the scatter plots of the data. Internal to the workings of the calculator are the estimated non-linear regressions with the productivity metric, CASE/FTE, estimated relative to caseload, violent crime rate, property crime rate, population served, type of jurisdiction (metro, regional, state), and interaction variables between these independent variables.

Laboratories testing the beta calculator were encouraged to share results with the Forensic Technology Center of Excellence (FTCoE) and Project FORESIGHT. Along with the results of the calculator, laboratories offered suggestions for the improvement of the workforce calculator. In addition to testing laboratories, we presented the beta calculator at a Western United States laboratory directors meeting and solicited suggestions for the updated calculator. Several of these suggestions guided the updates in the workforce calculator.

First, the sample laboratories used in the construction of the beta calculator resulted in some restrictions on personnel estimations because of statistical restrictions from in-sample caseloads. To overcome the statistical limitations, a greater number of large caseload laboratories was solicited to provide a wider range of estimates. Fortunately, the fiscal year 2018 and fiscal year 2019 Project FORESIGHT submissions expanded the caseload range to permit broader applicability of the model.

Second, several laboratory directors suggested that improved output would result if the personnel shortfall/surplus was broken down into operational staffing needs versus staffing needs for administration & support. This distinction is a featured output in the updated calculator.

Third, several laboratories encouraged an explicit note of the change in staffing suggested by the model. While such a change in FTE is a simple calculation, it was argued that having the explicit change noted through an independent source might offer greater assistance to local arguments for increased staffing.

The final change added to the model referenced the accompanying support for the capital budget. Several laboratory directors feared that a call for additional personnel lines would result in a directive to shift budgets from capital to personnel. Since the model estimation included an assumption that efficiency included the corresponding capital investments, the annual capital budget requirement to support caseload activity was added to the calculator output.

**Fig. 8:**
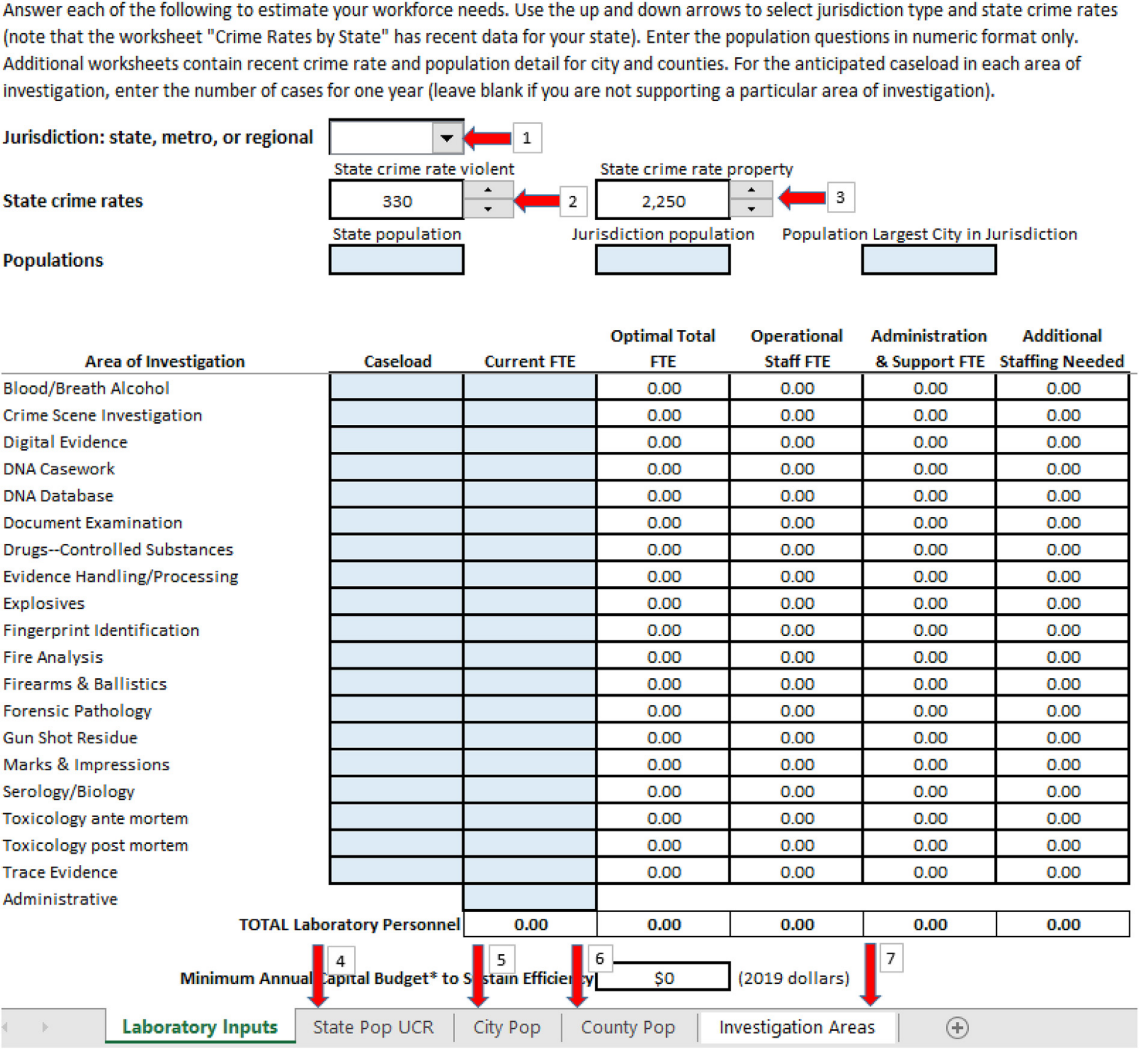
Key features of the workforce calculator.

The enhanced workforce calculator output incorporates the suggestions from the various test laboratories in their use of the beta workforce calculator. Fig. 8 shows the inputs and locations for the output.

A laboratory chooses from three drop down menus:

1. Type jurisdiction (state, metro, or regional)
2. State violent crime rate
3. State property crime rate

Next, the laboratory completes the population data for their state, jurisdiction, and the largest city within the jurisdiction. Additionally for each area of investigation, the laboratory enters the anticipated annual caseload in the relevant shaded areas. The laboratory optionally includes their current staffing. The output fields (non-shaded boxes) automatically populate as the laboratory enters caseloads and corresponding current FTE.

To assist in the input process, there are additional worksheets with U.S. Census Bureau data:

4. State populations (2015–2019) and Uniform Crime Reporting Program data (2018 & 2019).
5. City populations sorted by state (2015–2019).
6. County populations separated by state (2015–2019).

An additional worksheet includes some definitions:

7. The Project FORESIGHT definitions for each area of investigation.

**Fig. 9:**
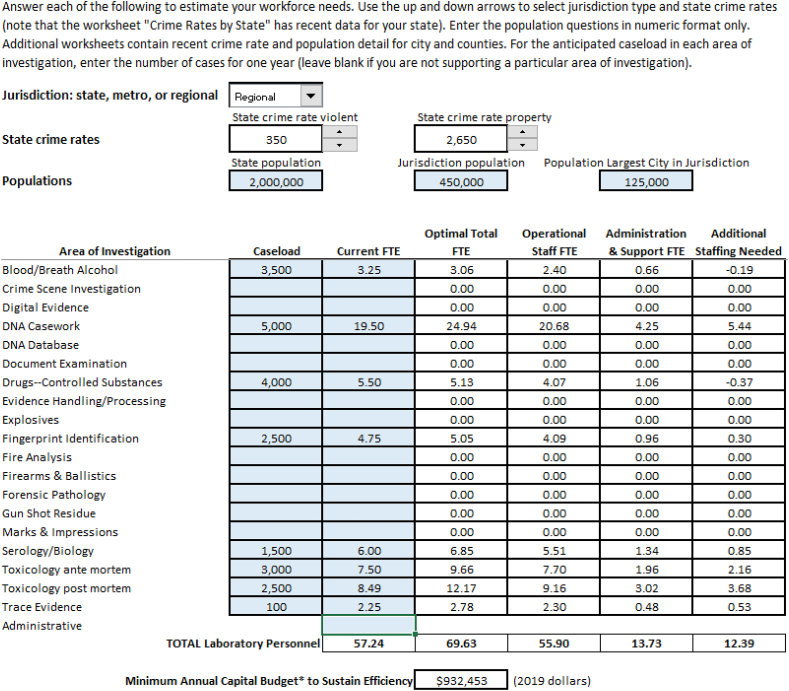
Example input/output.

Fig. 9 provides an example of the inputs and outputs for a hypothetical forensic laboratory.^6^ This hypothetical laboratory represents a typical regional laboratory covering a population of 450,000 with eight areas of investigation performed in the laboratory (Blood/Breath Alcohol, DNA Casework, Drugs-Controlled Substances, Fingerprint Identification, Serology/Biology, Toxicology ante mortem, Toxicology post mortem, and Trace Evidence Analysis). The output columns for Optimal 10.13039/501100007185Total FTE, Operational Staff FTE, and Administration & Support FTE are generated once the laboratory provides the detail on caseload. If the laboratory enters the current FTE column, the final output column, Additional Staffing Needed, will populate.

For the example laboratory, their current 57.24 FTE falls short by 12.39 FTE as the staffing required for optimal performance. The final output indicates the minimum annual capital investment to support that level of performance.

Although this sample laboratory represents a fictitious example for demonstration of the tool, consider the output from several trial laboratories. Twenty-eight laboratories shared results from their test of the updated calculator. Table 1 illustrates the recommended change in FTE from the workforce calculator for a representative laboratory from five jurisdictions: statewide, metropolitan, regional, regional with a city over 150,000 population within the region, and a medical examiner/coroner laboratory.

**Table 1:** Example laboratories recommended change in FTE.

Area of Investigation	Regional	Regional/metro	Metro	Coroner	Statewide
Blood/Breath Alcohol	−0.06	−0.22	0.25	−1.10	
Crime Scene Investigation			8.08		0.79
Digital Evidence			1.53		0.30
DNA Casework	−0.70	−1.97	0.42		9.25
Document Examination		0.37			
Drugs--Controlled Substances	1.83	−2.07	3.14		0.41
Fingerprint Identification	0.26	1.41	0.06		−0.99
Fire Analysis	0.36	0.26	0.08		0.57
Firearms & Ballistics	4.53	1.35	−0.14		0.48
Forensic Pathology				14.32	
Gun Shot Residue			−0.02		
Marks & Impressions	0.20				0.04
Serology/Biology		−0.18	−3.48		−0.15
Toxicology ante mortem					
Toxicology post mortem				−3.65	
Trace Evidence	0.32		−0.29		
**Increase/Decrease in FTE**	**6.73**	**−1.05**	**9.62**	**9.56**	**10.70**

Note that the FTE shortfall or surplus is relatively small in most areas of investigation, but the cumulative effect is substantial for many laboratories. The example regional laboratory has small deviations, both positive and negative across areas of investigation with the exception of firearms & ballistics with a need for over 4.5 FTE. The regional laboratory with a larger city within the region has modest differences from the efficient allocation of personnel and suggests a surplus employment of 1.05 FTE. The metropolitan laboratory shows a large shortfall of personnel, primarily in Crime Scene Investigation. The statewide laboratory also has modest deviations with the exception of a large shortfall in DNA Casework personnel. Finally, the medical examiner/coroner example laboratory appears slightly overstaffed in the toxicology areas, but dramatically understaffed in forensic pathology.

Beyond these example laboratories, we found that the average shortfall for laboratories that tested the calculator was 7.43 FTE.^7^ That average shortfall represented 19.7% of the current staffing of these laboratories.

## 7 Discussion

The forensic laboratory workforce calculator begins to address the forensic science macro-level shortfall identified by the 2019 NIJ Report to Congress on the needs of the forensic community [1]. The calculator permits estimation of laboratory-level needs based upon the staffing levels of high performers in the forensic science community. The current calculator is not the final answer. The inherent estimates underlying the calculator will need regular updates to reflect improvements in technology and scientific technique. Likewise, there are sure to be additional factors worthy of consideration. For example, if there is a consensus for inclusion of turnaround time and/or backlog considerations, then the calculator requires updates. Other considerations could include detail across characteristics of personnel including education, training, experience, diversity, and any other issues pertinent to forensic laboratories. Future versions of the calculator might provide greater detail across expenditures for personnel, capital, consumables, and overhead associated with alternative technologies and processes.

Additionally, the transition to the National Incident-Based Reporting System (NIBRS) may provide more information about the forensic laboratory staffing needs. Future versions of the forensic laboratory workforce calculator will require re-estimation to include NIBRS.

## 8 Concluding comments

All laboratories are encouraged to visit the Forensic Technology Center of Excellence site and try the Workforce Calculator [27]. The calculator is free to use. After trying the calculator, laboratories are encouraged to provide feedback to FTCoE or Project FORESIGHT, including comparison of their current staffing to the output of the workforce calculator. Additionally, all laboratories are encouraged to participate in Project FORESIGHT. As of this writing, participation in Project FORESIGHT continues to be free of charge to participating laboratories.

## Declaration of competing interest

The authors declare that they have no known competing financial interests or personal relationships that could have appeared to influence the work reported in this paper.

## Footnotes

1 This project was supported by Award No. 2016-MU-BX-K110, awarded by the National Institute of Justice, Office of Justice Programs, U.S. Department of Justice. The opinions, findings, and conclusions or recommendations expressed in this publication are those of the author and do not necessarily reflect those of the Department of Justice.

2 The definitions of each casework measure may be found in Houck et al. (2009) [14] and are included with the LabRAT data collection tool.

3 This maximization of processed cases subject to a budget constraint is equivalent to the minimization of the average cost of case processing, given the quality standards.

4 Toxicology ante mortem was chosen among the 21 areas of investigation for demonstration purposes only. Similar relationships exist for each area of investigation between each of the four ratios and caseload.

5 An average of 8.3% of FORESIGHT laboratories meet the 90% or higher efficiency standard each year.

6 The video demonstration with the initiation of the inputs and generation of the output is available via YouTube [28].

7 Remember that these 28 laboratories do not represent a random sample. They are a convenience sample of voluntary laboratories and have a heavy representation from ASCLD Maximus award laboratories.
